# Supplementary material for: Assessing port service quality: An application of the extension fuzzy AHP and importance-performance analysis
Source: PLoS One. 2022 Feb 25;17(2):e0264590. doi: 10.1371/journal.pone.0264590 (PMC8880942; doi:10.1371/journal.pone.0264590)
Supplement: S1 File — (ZIP) [file pone.0264590.s001.zip › Supporting information.docx]

**Table 1. Descriptive statistics for constructs and critera for the satisfaction measurement:**

|  | Mean | Standard Deviation | Min | Max | Standared Error |
| --- | --- | --- | --- | --- | --- |
| Constructs | 16.67 | 3.19 | 12.28 | 20.96 | 0.53 |
| Local weight | 20.69 | 4.75 | 14.22 | 32.09 | 0.16 |
| Global weight | 3.45 | 0.92 | 2.18 | 5.25 | 0.03 |

**Table 2. Descriptive statistics for constructs and critera for the importance measurement**

|  | Mean | Standard Deviation | Min | Max | Standared Error |
| --- | --- | --- | --- | --- | --- |
| Constructs | 16.67 | 3.00 | 12.53 | 21.07 | 0.50 |
| Local weight | 20.69 | 5.26 | 12.30 | 33.28 | 0.18 |
| Global weight | 3.45 | 0.92 | 1.96 | 5.51 | 0.03 |

**Table 3. The importance and satisfactioin measurement**

| Subcriteria | Local weights in the second-order (%) | |
| --- | --- | --- |
|  | Importance weight | Satisfaction weight |
| TA1 | 23.12 | 31.23 |
| TA2 | 34.23 | 11.34 |
| TA3 | 16.23 | 23.45 |
| TA4 | 12.09 | 23.06 |
| TA5 | 14.33 | 10.92 |
| RL1 | 27.63 | 30.65 |
| RL2 | 29.72 | 22.34 |
| RL3 | 18.68 | 15.45 |
| RL4 | 23.97 | 31.56 |
| AS1 | 20.09 | 12.34 |
| AS2 | 30.99 | 19.45 |
| AS3 | 23.43 | 23.44 |
| AS4 | 11.00 | 10.12 |
| AS5 | 6.34 | 23.09 |
| AS6 | 8.15 | 11.56 |
| EM1 | 11.23 | 9.34 |
| EM2 | 17.47 | 16.47 |
| EM3 | 23.56 | 21.23 |
| EM4 | 34.11 | 18.89 |
| EM5 | 13.63 | 34.07 |
| RP1 | 21.56 | 34.12 |
| RP2 | 31.45 | 24.76 |
| RP3 | 16.98 | 20.91 |
| RP4 | 30.01 | 20.21 |
| DI1 | 34.11 | 13.24 |
| DI2 | 21.23 | 17.34 |
| DI3 | 19.01 | 23.67 |
| DI4 | 17.19 | 21.38 |
| DI5 | 8.46 | 24.37 |

**Figure 1. The IPA results**
