# Supplementary material for: Assessing port service quality: An application of the extension fuzzy AHP and importance-performance analysis
Source: PLoS One. 2022 Feb 25;17(2):e0264590. doi: 10.1371/journal.pone.0264590 (PMC8880942; doi:10.1371/journal.pone.0264590)
Supplement: Attachment — Submitted filename: Response to reviewers.docx [file pone.0264590.s002.docx]

**Reviewer #1:**

Thank you for inviting me to review this interesting article. I realized that it is a very important study for assessing port service quality. However, the following points need to be improved before publication.

1. Rationale of the topic and more detailed results of the present work should be given in the abstract section.

Answer: Thanks for your comments, we revised the manuscript in the red-colored text (Seeing rows 25-27 and rows 32-42)

2. Why triangular fuzzy numbers are preferred should be emphasized. The rationale for the methods used must be clearly stated in the introduction sections of the study. Because there are a lot of MCDM algorithm in the literature. Try to refer more recent MCDM articles-related (i.e., port, logistics service, 3PL, etc.), for this purpose, the following article are suggested:

10.1109/ACCESS.2021.3121607

10.3390/axioms10020048

10.3390/axioms10010034

10.3390/math9080886

Answer: Thanks for your comments, we revised the manuscript in the red-colored text (Seeing rows 73-83)

3. Why 29 criteria are considered should be explained?

Answer: Thanks for your comments, we revised the manuscript in the red-colored text (Seeing rows 222-231)

4. Providing more information about experts would be better.

Answer: Thanks for your comments, we revised the manuscript in the red-colored text (Seeing Table 4)

5. Which aggregation operator are used while combining expert judgments?

Answer: Thanks for your comments, we explain as follow:

As seen in the article, we successfully surveyed 15 respondents. Thence, for each dimension (i.e., the RL dimension), we can form 15 individual judgment matrixes. Next, such 15 matrices are integrated together to establish a fuzzy positive reciprocal matrix via the formula Keep in mind that this aggregation operator is employed commonly in the prior literature, for instance, Wang, Dang, and Nguyen (2021), Wang, Dang, and Hsu (2021), etc. Please seeing rows 304-310.

6. It is recommended to write the managerial impact section.

Answer: Thanks for your comments, we revised the manuscript in the red-colored text (Seeing rows 486-508)

7. The manuscript requires minor revision by native speakers.

Answer: Thanks for your comments, we had one English speaker revise the manuscript.

Reference:

Wang, C. N., Dang, T. T., & Nguyen, N. A. T. (2021). Outsourcing reverse logistics for e-commerce retailers: A two-stage fuzzy optimization approach. *Axioms*, *10*(1), 34.

Wang, C. N., Dang, T. T., & Hsu, H. P. (2021). Evaluating sustainable last-mile delivery (LMD) in B2C E-commerce using two-stage fuzzy MCDM approach: A case study from Vietnam. *IEEE Access*, *9*, 146050-146067.

**P.S.**

**The authors are very grateful to the reviewer to check the paper so carefully and propose so many constructive suggestions to improve the paper. Many thanks!**

**Reviewer #2:**

This study aims to investigate port service quality, then a real study case is presented. The paper is quite well organised and the contribution is quite well positioned in the current background.

1. The introduction section should more in depth clarify that they refer to container ports however the methodology can be used also for other types of port. For example, can it be used for short-sea shipping one?

Answer: Thanks for your comments, we revised the manuscript in the yellow-highlighted text (Seeing rows 47-51). Besides, it can be confirmed that the methodology in this paper can be applicable for short-sea shipping ports.

2. The authors are suggested to detail the method proposed giving some useful info also for readers not so experts as they are.

Answer: Thanks for your comments, we revised our manuscript in the red-colored texts (Seeing rows 73-83). Note that this comment is relatively consistent with that of the other reviewer.

3. Fig.2's description needs to follow closer the framework proposed, clarifying each steps pictured. Besides, the description of methods (subsections of section 3) could follow the research framework presented in Fig. 2. For example, each subsection title could be equal to box in Figure 2.

Answer: Thanks for your comments, we explained as follow:

First of all, we revised the manuscript in the yellow-highlighted text (Seeing rows 205-216).

Secondly, we also presented our paper corresponding to the flowchart in Figure 2. More specifically, Step 1 is displayed in Section 3.2, whilst Step 2 is exhibited in Section 3.5. And Step 3 is presented in Section 3.6.

4. Formulas are not legible, e.g. eq. 2 and 3, 4 and so on. Symbols are also not legible in the main text (e.g. see row 294).

Answer: Thanks for your comments. We checked all formulas and mathematical symbols throughout our article to make sure that they are all legible.

**P.S.**

**The authors are very grateful to the reviewer to check the paper so carefully and propose so many constructive suggestions to improve the paper. Many thanks!**
